# *Lactobacillus casei* relieves liver injury by regulating immunity and suppression of the enterogenic endotoxin‐induced inflammatory response in rats cotreated with alcohol and iron

**DOI:** 10.1002/fsn3.2486

**Published:** 2021-08-11

**Authors:** Xuelong Li, Jianmin Han, Ying Liu, Hui Liang

**Affiliations:** ^1^ Department of Human Nutrition College of Public Health Qingdao University Qingdao China; ^2^ Department of Clinical Nutrition The Affiliated Yantai Yuhuangding Hospital of Qingdao University Yantai China; ^3^ Basic Medical College Qingdao University Qingdao China

**Keywords:** alcohol and iron, immunity, inflammatory, *Lactobacillus casei*, liver injury

## Abstract

Excessive alcohol and iron intake can reportedly cause liver damage. In the present study, we investigated the effect of *Lactobacillus casei* on liver injury in rats co‐exposed to alcohol and iron and evaluated its possible mechanism. Sixty male Wistar rats were randomly divided into three groups for 12 weeks: the Control group (administered normal saline by gavage and provided a normal diet); alcohol +iron group (Model group, treated with alcohol [3.5–5.3 g/kg/day] by gavage and dietary iron [1,500 mg/kg]); Model group supplemented with *L. casei* (8 × 10^8^ CFU kg^−1^ day^−1^) (*L. casei* group). Using hematoxylin and eosin (HE) staining and transmission electron microscopy, we observed that *L. casei* supplementation could alleviate disorders associated with lipid metabolism, inflammation, and intestinal mucosal barrier injury. Moreover, levels of serum alanine aminotransferase, gamma‐glutamyl transferase, triglyceride (TG), and hepatic TG were significantly increased in the model group; however, these levels were significantly decreased following the 12‐week *L. casei* supplementation. In addition, we observed notable improvements in intestinal mucosal barrier function and alterations in T lymphocyte subsets and natural killer cells in *L. casei*‐treated rats when compared with the model group. Furthermore, *L. casei* intervention alleviated serum levels of tumor necrosis factor‐α and interleukin‐1β, accompanied by decreased serum endotoxin levels and downregulated expression of toll‐like receptor 4 and its related molecules MyD88, nuclear factor kappa‐B p65, and TNF‐α. Accordingly, supplementation with *L. casei* could effectively improve liver injury induced by the synergistic interaction between alcohol and iron. The underlying mechanism for this improvement may be related to immune regulation and inhibition of enterogenic endotoxin‐mediated inflammation.

## Graphical abstract

*Lactobacillus casei* could effectively improve liver injury induced by co‐exposure to alcohol and iron. The mechanism may be related to the immune regulation and enterogenic endotoxin‐mediated inflammation inhibition.

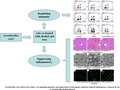

## 1 INTRODUCTION

The liver is an essential organ for alcohol metabolism and iron storage; therefore, it is the primary target organ for alcohol injury and iron overload (Lainé et al., 2017; Lakhal‐Littleton et al., 2016). The intake of both excessive alcohol and iron (iron or iron‐rich foods) can lead to liver damage via oxidative stress and lipid peroxidation (Osna et al., 2017; Pietrangelo, 2016); excessive alcohol intake can also promote iron absorption, resulting in excessive iron deposition in the liver (Corradini & Pietrangelo, 2012; Grochowski et al., 2019). In addition, studies have revealed that co‐exposure to alcohol and iron results in synergistic oxidation and cumulative effects that aggravate hepatocyte injury (Fletcher & Powell, 2003; Sumida et al., 2001).

Reportedly, the immune status of T lymphocytes is closely related to the occurrence and development of alcoholic liver injury (Matos et al., 2013). Long‐term drinking can inhibit innate immune cells and reduce the number and activity of lymphocyte subsets (Støy et al., 2015). Alcohol consumption also impacts the functions of hepatic natural killer (NK) cells (Cui et al., 2017). In addition, disturbances in iron homeostasis have been associated with altered immune functions and liver injury. Iron overload can affect lymphocyte proliferation/maturation, induce the apoptosis of T lymphocytes, and selectively affect peripheral T lymphocytes, with hepatocellular ballooning injury (Buracco et al., 2017; Handa et al., 2016). Based on these previous reports, it can be postulated that the immune state caused by excessive alcohol and iron intake mediates the development of liver injury.

Endotoxins are a major component of Gram‐negative bacterial cell walls (Clementi et al., 2017). Notably, excessive alcohol intake is known to result in enhanced intestinal mucosal permeability, which, in turn, causes the leakage of large amounts of gut‐derived endotoxins from the gut lumen into the systemic circulation (Meroni & Longo, 2019). These endotoxins enter the liver through the portal vein and bind to toll‐like receptor 4 (TLR4) on the Kupffer cell surface. Subsequently, a cascade reaction triggers downstream signaling molecules, including MyD88 and nuclear factor kappa‐B (NF‐κB), resulting in the activation of Kupffer cells to secrete and release excessive proinflammatory cytokines such as tumor necrosis factor (TNF)‐α and interleukin (IL)‐1β (Li et al., 2012; Zannetti et al., 2016), which ultimately causes inflammatory injury to hepatocytes. Additionally, excessive iron uptake by macrophages can enhance NF‐κB activity, further promoting inflammatory factor production and intensifying liver injury (Liu Meng‐na & Zhang, 2019).

Probiotics are living microorganisms that are beneficial to the health of the host. Appropriate probiotic supplementation improves the repair of intestinal mucosal damage (Deng et al., 2017) and affords anti‐inflammatory (Li et al., 2016), antioxidant (Wang et al., 2017), and immune regulation (La Fata et al., 2018). As a well‐known probiotic, *Lactobacillus casei*, in addition to the above characteristics, has attracted extensive attention, especially for its protective effect against alcoholic liver injury. Several studies have achieved good therapeutic effects following *L*. *casei* supplementation in patients with alcoholic cirrhosis (Koga et al., 2013; Macnaughtan et al., 2020; Stadlbauer et al., 2008), during which iron deposition is a known marker (Zhang & Krinsky, 2004). Currently, the mechanism underlying the protective effect of *L*. *casei* on alcoholic liver injury remains poorly understood. Accordingly, we aimed to explore the effects of *L*. *casei* on liver injury in rats induced by the synergistic interaction between alcohol and iron. Furthermore, we investigated the mechanism underlying immune regulation and inhibition of enterogenic endotoxin‐mediated inflammation.

## 2 MATERIALS AND METHODS

### 2.1 Animals and ethics statement

Adult male Wistar rats (180–220 g, aged 2 months) were provided by the Animal Experiment Centre (Qingdao, China). The experimental protocol was approved by the Animal Ethics Committee of Qingdao University.

### 2.2 Experimental design

After one week of adaptive feeding, 60 male Wistar rats were randomly divided into 3 groups (20 animals/group): the control group, normal saline by oral gavage and a standard diet; the Model group (alcohol +iron group), fed a standard diet containing high dietary iron (1,500 mg/kg) and 56% v/v alcohol by oral gavage (3.5g kg^−1^ day^−1^ 2 weeks +5.3g kg^−1^ day^−1^ 10 weeks); the *L. casei* group (*L. casei* treatment group), fed a standard diet containing high dietary iron (1,500 mg/kg) and oral alcohol and *L. casei* (the dose of alcohol was the same as in the model group; *L. casei*, 8 × 10^8^ CFU/kg^−1^ day^−1^). The experiment was performed over a 12‐week period.

*Lactobacillus casei* was provided by Yangle Duo China Investment Co., Ltd. (*L. casei* content ≥1 × 10^8^ CFU/ml). The alcohol (56% [v/v] ethanol) used was Red Star Erguotou (Beijing Red Star Co., Ltd., Beijing, China) (Ma et al., 2018).

After 12 weeks, the animals were sacrificed, and the blood (serum or plasma), liver, and small intestinal tissues were harvested and stored at −80°C. Fresh liver tissues were quickly excised, fixed in 10% formaldehyde, and embedded in paraffin.

### 2.3 Determination of serum iron (SI), liver iron concentration (LIC), inflammatory factors, endotoxin, liver function, and lipid metabolism

The levels of SI and LIC were measured using inductively coupled plasma mass spectrometry (ICP‐MS) by strictly following the manufacturer's instructions. In addition, the levels of interleukin‐1β (IL‐1β), tumor necrosis factor‐α (TNF‐α), and endotoxin were measured by a competitive enzyme immunoassay using an enzyme‐linked immunosorbent assay (ELISA) kit (Cloud‐Clone Corp, Katy, USA), in accordance with the manufacturer's instructions.

Serum levels of alanine aminotransferase (ALT), aspartate aminotransferase (AST), gamma‐glutamyltransferase (GGT), total cholesterol (TC), and triglyceride (TG), as well as hepatic TG levels, were measured using commercially available kits on a HITACHI 7020 chemistry analyzer.

### 2.4 Histopathological analysis

The formalin‐fixed liver tissues were subjected to hematoxylin–eosin (HE) staining, and morphological changes were examined under a light microscope (Olympus BX60, Tokyo, Japan). Ultrastructures of the small intestinal and liver tissues were observed using a JEM‐1200EX transmission electron microscope (TEM; JEOL, Tokyo, Japan).

### 2.5 Detection of T lymphocyte subsets and NK cells

The number of CD4^+^, CD8^+^ T lymphocyte subsets, and NK cells in the peripheral blood of rats was determined flow cytometrically. Monoclonal antibodies labeled with fluorochrome, TCRαβ‐FITC, CD3‐FITC, CD4‐PE, and CD8‐PE were procured from BD Pharmingen. For each test, 100 μl of fresh heparinized rat whole blood was incubated with indicated antibodies for 15 min, then lysed with FACS™ lysing solution, washed with phosphate‐buffered saline, fixed, and eventually detected by BD FACSAria with DB FACSDiva software.

### 2.6 Small intestine tracer permeability assay

In brief, the ends small intestinal middle segment tissues (2 cm) were ligated, and 2 mg/ml EZ‐link Sulfo‐NHS‐Biotin was slowly injected; this was followed by incubation for 5 min at room temperature. The tissues were fixed in 4% paraformaldehyde. After 24 hr, the sections were washed three times with phosphate‐buffered saline, embedded in paraffin, and cut into 5‐μm thick sections. The sections were incubated with streptavidin DyLightTM 488 conjugated (1:500 dilution) for 30 min in the dark. The distribution of EZ‐link Sulfo‐NHS‐biotin was observed using a fluorescence microscope.

### 2.7 Expressions of TLR4 and MyD88 signaling pathway‐related proteins by Western blot analysis

Proteins were extracted from liver tissues using the Nuclear and Cytoplasmic Protein Extraction Kit (Beyotime, Jiangsu, China), in accordance with the manufacturer's instructions. Total protein content was determined using the BCA Protein Assay Kit (Beyotime). Equal amounts of protein were subjected to sodium dodecyl sulfate‐polyacrylamide gel electrophoresis (SDS‐PAGE) on 5% to 10% polyacrylamide gels and then transferred onto PVDF membranes (Millipore, Bedford, MA). The membranes were blocked with 10% nonfat milk in TBST (Tris‐buffered saline, 0.1% Tween 20) and incubated with anti‐NF‐κB p65 and anti‐IκB (Cell Signalling Technology, Danvers, MA.), anti‐histone H3, and anti‐β‐actin (1:1,000 dilution; Proteintech, Rosemont, IL) antibodies at 4°C overnight. Subsequently, membranes were washed three times with TBST for 10 min, followed by incubation with the corresponding secondary antibodies (1:3,000 dilution; Zhongshan Goldenbridge, Beijing, China) for 2 hr at 37°C. Finally, protein bands were visualized using an enhanced chemiluminescence detection kit (Beyotime). Histone H3 and β‐actin served as internal controls for nucleoprotein and cytoplasmic proteins, respectively.

### 2.8 Statistical analysis

All data values are expressed as mean ±standard deviation using SPSS (version 18; IBM Corp., Chicago, IL, USA). One‐way analysis of variance (ANOVA) was used to compare multiple groups, followed by Duncan's multiple range test. *p* ˂ 0.05 was deemed statistically significant.

## 3 RESULTS

### 3.1 Effects of *L. casei* on body weight

**FIGURE 1:**
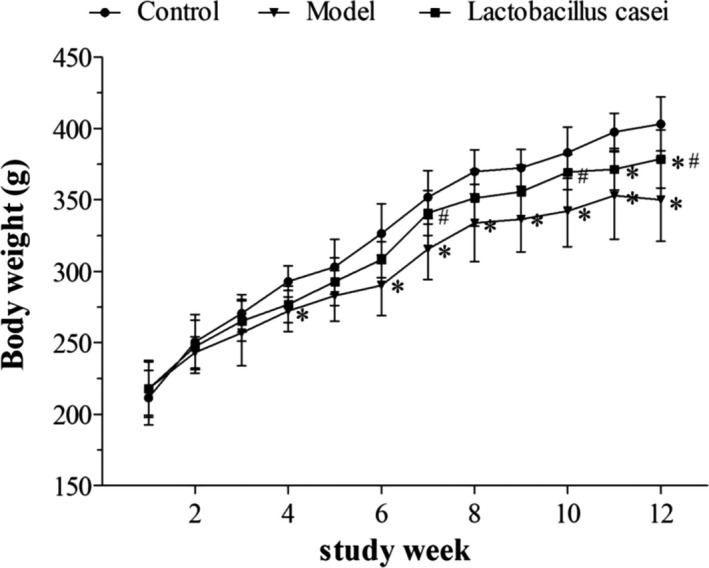
Effects of *L. casei* on body weight. Control: the control group; Model: the model group; *Lactobacillus casei*: the *L. casei* group. ^*^ *p* < .05 versus the control group, ^#^ *p* < .05 versus the model group

The recorded body weights before and after the intervention are shown in Figure 1. No significant difference in body weight was observed between the groups before intervention (*p* > .05). However, the weight of the model group reached (348.17 ± 29.18) g after the 12‐week intervention, which was significantly decreased when compared with the control group (401.17 ± 18.93) g (*p* < .05). *Lactobacillus casei* supplementation significantly improved the weight loss observed in rats cotreated with alcohol and iron (*p* <.05) (Figure 1).

### 3.2 Effects of *L. casei* on SI and LIC

**FIGURE 2:**
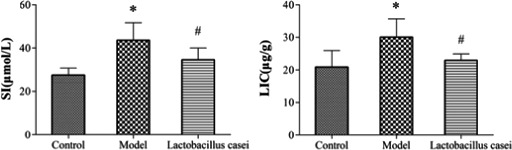
Effects of *L. casei* on serum iron and liver iron concentration. SI: serum iron; LIC: liver iron concentration; Control: the control group; Model: the model group; *Lactobacillus casei*: the *L. casei* group. ^*^ *p* < .05 versus the control group, ^#^ *p* < .05 versus the model group

As shown in Figure 2, SI and LIC were significantly increased (58.17% and 24.46%, respectively) in the model group when compared with those in the control group (*p* < .05); these levels were significantly decreased following *L. casei* supplementation by 20.60% and 24.65%, respectively (*p* < .05).

### 3.3 Effects of *L. casei* on pathological changes in the liver

**FIGURE 3:**
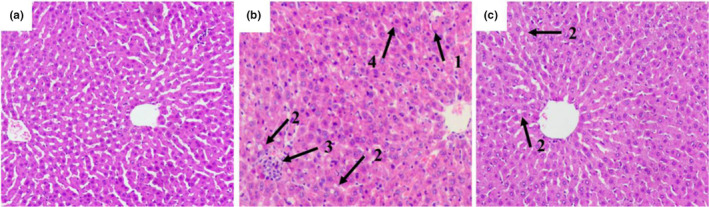
Effects of *L. casei* on pathological changes in the liver (Hematoxylin–eosin [HE] Staining, ×200). 1: Mallory body; 2: fat vacuoles; 3: inflammatory cell infiltration; 4: vitreous degeneration of hepatocytes. (a) The control group; (b) the model group; (c) the *L*. *casei* group

We examined liver sections from each group using HE staining and light microscopy. The control group presented a typical lobular structure, with an ordered hepatic cord, normal hepatocyte structure, and the absence of steatosis or inflammatory infiltration (Figure 3a). Liver damage was observed in the model group, with hepatic cord derangement, liver cell swelling, inflammatory infiltration, microvesicular steatosis, specific Mallory bodies, and fat vacuoles (Figure 3b). Compared with the model group, *L. casei* supplementation significantly ameliorated these histopathological changes and alleviated steatosis in the liver. In the *L. casei* group, the hepatic lobular structure was typical, the hepatic cord was orderly, hepatocyte swelling was alleviated, and only a few fat vacuoles were observed (Figure 3c).

**FIGURE 4:**
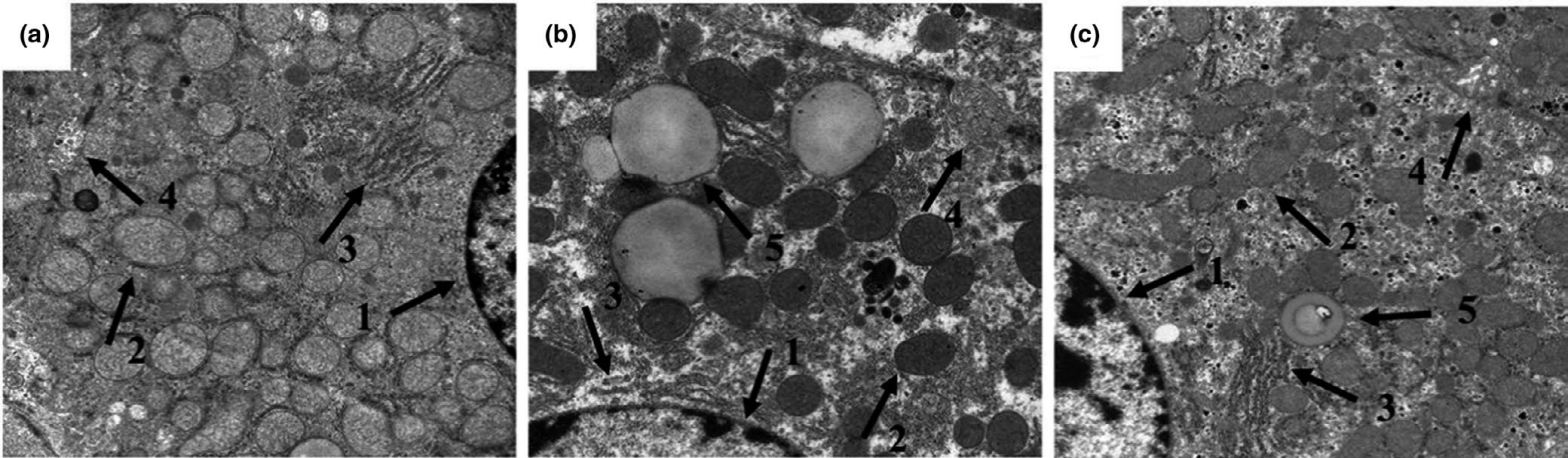
Effects of *L. casei* on ultrastructure changes in the liver (transmission electron microscopy, ×15,000). 1: nucleus; 2: mitochondria; 3: endoplasmic reticulum; 4: bile canaliculi; 5: lipid droplets. (a) The control group; (b) the model group; (c) the *L. casei* group

The control group displayed a normal mitochondrial structure, with clearly visible cristae, regularly arranged endoplasmic reticulum, and typically shaped bile canaliculi (Figure 4a). In the model group, large amounts of lipid droplets were observed. The bile canaliculi and microvilli were swollen, and the mitochondrial ridge was slightly blurred. In addition, the endoplasmic reticulum was arranged in a disorderly fashion (Figure 4b). Supplementation with *L*. *casei* alleviated the swelling in the microvilli and bile canaliculi. Structurally, mitochondria and endoplasmic reticulum tended to be normal, with only a few lipid droplets observed (Figure 4c). This indicated that *L. casei* supplementation could alleviate the abnormal liver tissue ultrastructure induced by cotreatment with alcohol and iron in rats.

### 3.4 Effects of *L. casei* on liver function and lipid metabolism

**FIGURE 5:**
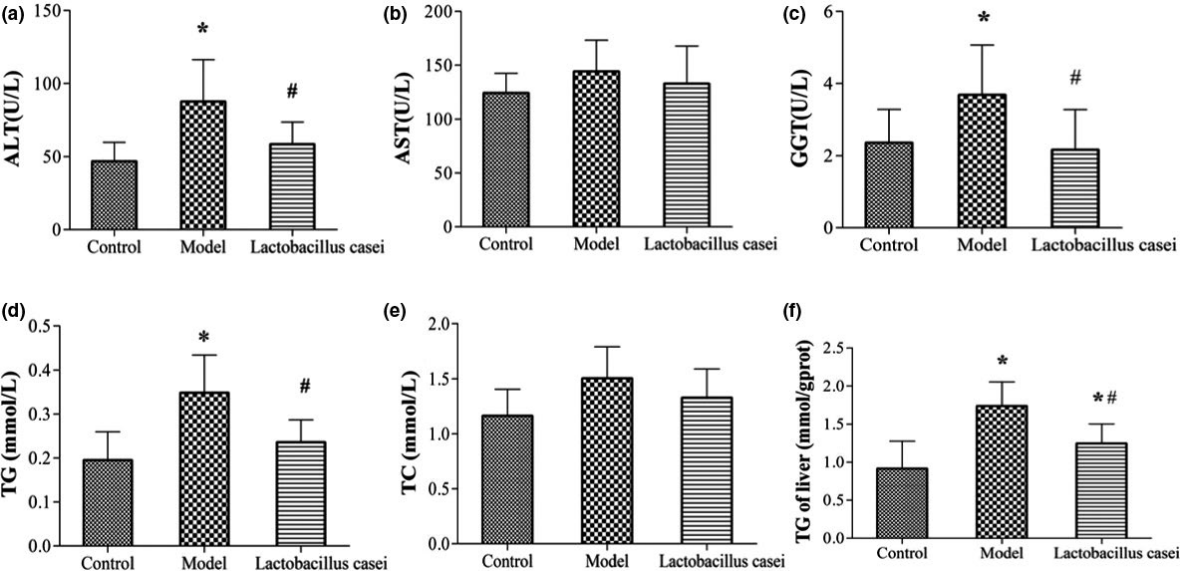
Effects of *L. casei* on liver function and lipid metabolism. ALT: alanine aminotransferase; AST, aspartate aminotransferase; GGT, gamma‐glutamyltransferase; TC, total cholesterol; TG, triglyceride. Control: the control group; Model: the model group; *Lactobacillus casei*: the *L. casei* group. ^*^ *p* < .05 versus the control group, ^#^ *p* < .05 versus the model group

As shown in Figure 5, the serum levels of ALT, GGT, TG, and hepatic TG were increased by 116.7%, 50.42%, 78.97%, and 86.08%, respectively, in the model group when compared with those in the control group (*p* < .05); these levels were significantly decreased by 33.33%, 38.59%, 32.38%, and 28.31%, respectively, (*p* < .05) after *Lactobacillus casei* supplementation.

### 3.5 Effects of *L. casei* on T lymphocyte subsets and NK cells

**FIGURE 6:**
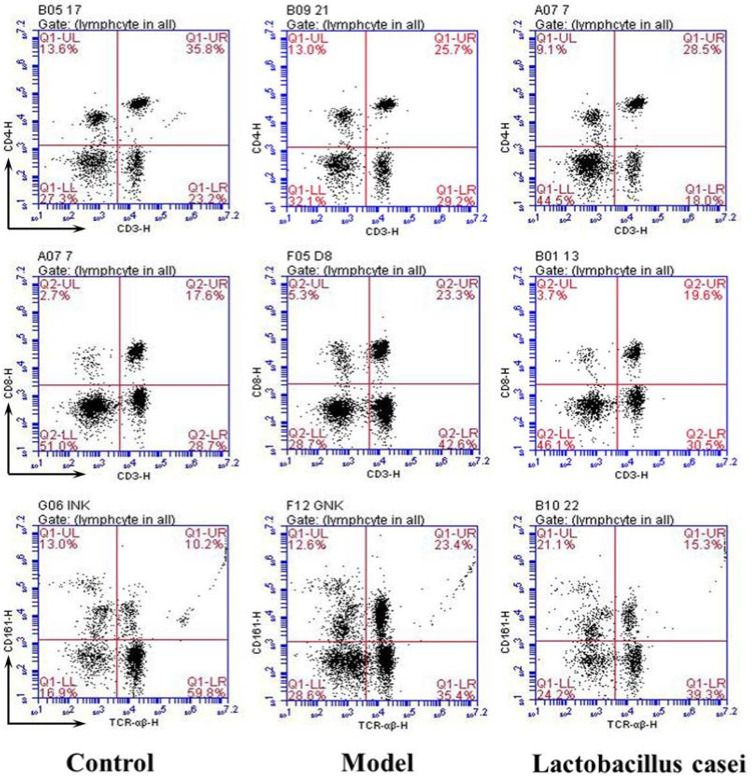
Effects of *L. casei* on T lymphocyte subsets and NK cells. Control: the control group; Model: the model group; *Lactobacillus casei*: the *L*. *casei* group

We observed that the CD4^+^ lymphocyte percentage and the CD4^+^/CD8^+^ ratio in the model group were significantly decreased when compared with the control group, while the CD8^+^ lymphocyte percentage and NK cell lymphocyte percentage were significantly increased (*p* < .05). *Lactobacillus casei* supplementation significantly decreased the percentage of CD8^+^ lymphocytes and NK cells (*p* < .05); simultaneously, the CD4^+^/CD8^+^ ratio was significantly increased (*p* < .05). Accordingly, *L. casei* supplementation could effectively improve the immune response in rats cotreated with alcohol and iron (Figure 6, Table 1).

**TABLE 1:** Proportion of T lymphocyte subsets and NK cells in peripheral blood of rats

Group	CD3^+^CD4^+^ lymphocytes (%)	CD3^+^CD8^+^ lymphocytes (%)	CD4^+^/CD8^+^ ratio	TCRαβ^+^ CD161^+^ NK lymphocytes (%)
Control	35.90 ± 2.15	18.03 ± 1.11	1.99 ± 0.04	11.00 ± 0.98
Model	25.90 ± 1.71*	23.90 ± 1.97*	1.08 ± 0.02*	23.90 ± 2.29*
*Lactobacillus casei*	27.73 ± 1.78*	20.27 ± 2.08^#^	1.41 ± 0.17^*#^	15.63 ± 1.72^*#^

Note

Control: the control group; Model: the model group; *Lactobacillus casei*: *L*. *casei* group.

* *p* < .05 versus the control group.

^#^ *p* < .05 versus the model group.

### 3.6 Effects of *L. casei* on intestinal permeability

#### 3.6.1 Effects of *L. casei* on the small intestinal tracer permeability

**FIGURE 7:**
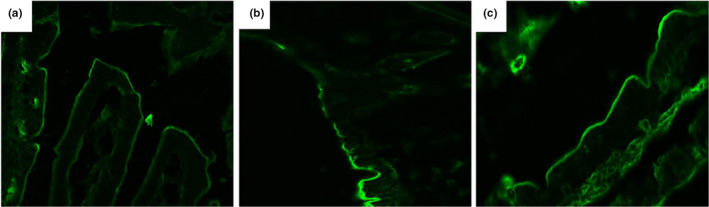
Effects of *L. casei* on the small intestinal tracer permeability (×400). (a) The control group; (b) the model group; (c) the *L. casei* group

In the control group, the fluorescent tracer was continuous and mostly confined to the intestinal lumen of the small intestine (Figure 7a). In the model group, the fluorescent tracer lacked continuity, demonstrating varying degrees of infiltration (Figure 7b). We observed that intestinal permeability was effectively improved by *L. casei* supplementation (Figure 7c).

#### 3.6.2 Effects of *L. casei* on tight junctions of intestinal epithelia

**FIGURE 8:**
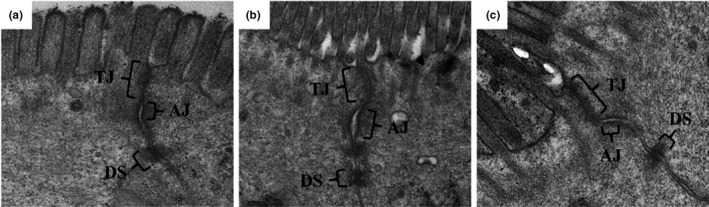
Effects of *L. casei* on tight junctions of intestinal epithelia (transmission electron microscope ×50,000). (a) The control group; (b) the model group; (c) the *L. casei* group. TJ, tight junctions; AJ, adherens junctions; DS, desmosomes

The control group revealed a normal ultrastructure of the intercellular junction of epithelial cells (Figure 8a). Compared with the control group, the tight junctions and adherens junctions in the model group showed a widened gap, with a reduced electron density (Figure 8b). *Lactobacillus casei* supplementation improved the intercellular junctional structures in the small intestine tissue, narrowing the gap closer to that observed in the control group (Figure 8c).

#### 3.6.3 Effects of *L. casei* on endotoxin

**FIGURE 9:**
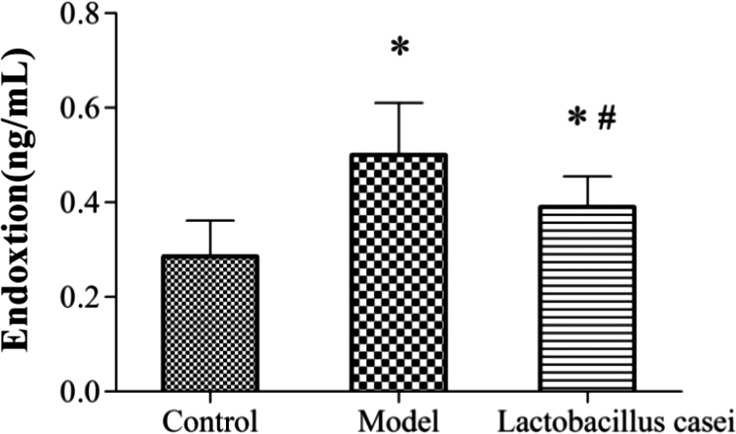
Effects of *L. casei* on endotoxin. Control: the control group; Model: the model group; *Lactobacillus casei*: the *L. casei* group. ^*^ *p* < .05 versus the control group, ^#^ *p* < .05 versus the model group

As shown in Figure 9, the serum level of endotoxin in the model group was significantly increased by 72.41% when compared with that in the control group (*p* < .05); however, this level was significantly decreased by 22% (*p* < .05) following *L. casei* supplementation.

### 3.7 Effects of *L. casei* on TLR4 signaling pathway and inflammation

**FIGURE 10:**
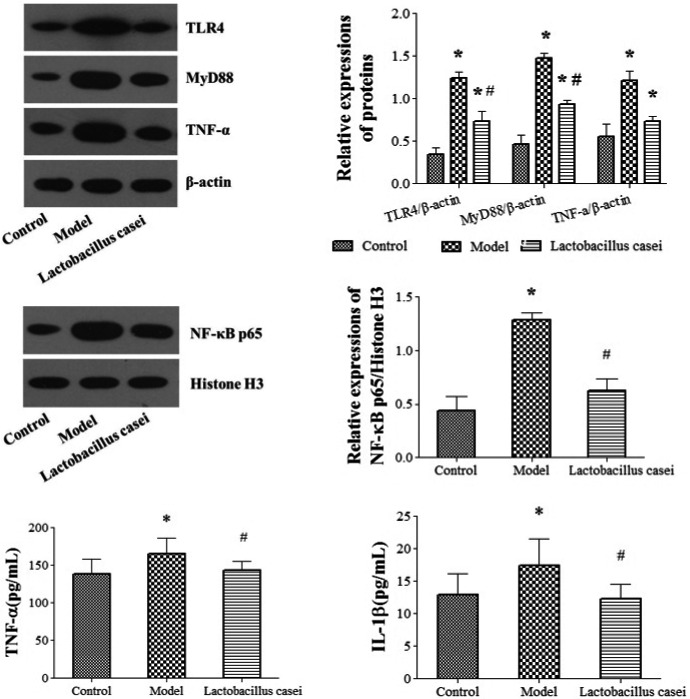
Effects of *L. casei* on TLR4 signaling pathway and inflammation. TNF‐α, tumor necrosis factor‐α; IL‐1β, interleukin‐1β. Control: the control group; Model: the model group; *Lactobacillus casei*: the *L. casei* group. ^*^ *p* < .05 versus the control group, ^#^ *p* < .05 versus the model group

As shown in Figure 10, the expression of TLR4, MyD88, TNF‐α, and NF‐κB p65 proteins was upregulated following cotreatment with alcohol and iron when compared with the control group (*p* < .05). Notably, the expression of TLR4, MyD88, TNF‐α, and NF‐κB p65 was downregulated following *L. casei* supplementation by 39.84%, 34.25%, 38.10%, and 51.16%, respectively (*p* < .05).

The serum levels of TNF‐α and IL‐1β in the model group were significantly increased by 25.28% and 27.36% when compared with those in the control group (*p* < .05); these levels were significantly decreased by 14.36% and 30.17%, respectively (*p* < .05), following *L. casei* supplementation.

## 4 DISCUSSION

Epidemiological studies have indicated that excessive intake of alcohol and iron can damage liver functions. Alcohol is a leading cause of liver diseases worldwide (Leggio & Lee, 2017). The number of proliferating hepatocytes was significantly increased in mice fed an excess‐iron diet, with the iron overload reportedly inducing mitochondrial injury (Furutani et al., 2006). Even under low alcohol intake, a certain amount of iron overload can cause significant liver damage (Gao et al., 2017). In the present study, a rat model of liver injury was established by co‐administering alcohol (by gavage) and a high‐iron feed, and the impact of *L. casei* supplementation was evaluated. Our study demonstrated that *L. casei* supplementation affords superior protection against liver injury in rats induced by the synergistic interaction between alcohol and iron.

Herein, we observed that *L. casei* significantly improved the weight of rats treated with alcohol plus iron, indicating that nutritional intake and absorption in rats were affected. This finding is consistent with our previous report (Ma et al., 2018). One possible explanation is damage to liver function. In addition, serological and pathological examinations indicated that the liver damage caused by combined exposure to alcohol and iron, including liver function decline, lipid metabolism disorders, and inflammatory cell infiltration, were significantly alleviated by *L. casei* supplementation, with a significant reduction in SI and MIC. These results indicate that *L. casei* could effectively improve liver injury in rats induced by the synergistic interaction between alcohol and iron.

Previous studies have revealed that liver injury is closely associated with immune system disorders. Matos et al. have reported that T lymphocytes are closely related to the occurrence and development of alcoholic liver injury, with all patients with alcoholic liver disease presenting decreased lymphocyte counts (Matos et al., 2013). Annie et al. have observed that NK cells play a crucial role in regulating chronic inflammatory diseases by modulating the balance between liver inflammation and cell repair (Annie et al., 2017). Reportedly, NK cell depletion can improve hepatic injury and survival (Kawabata et al., 2008; Qu et al., 2015). In addition, studies have shown that iron overload is closely related to the immune system. Chen et al. have revealed that iron overload induces T lymphocyte apoptosis and decreases the percentage of CD3^+^ T cells while increasing the percentage of regulatory T (Treg) cells and the ratio of CD4/CD8 (Chen et al., 2017). Previous reports have also demonstrated that *L. casei* plays a role in regulating immunity. Vaisberg et al. have documented that *L. casei* can modulate systemic and airway immune responses postmarathon (Vaisberg & Paixão, 2019). Aktas et al. have reported that *L. casei* can alter the gut microbiota composition and modulate the host immune response (Aktas et al., 2016). Song et al. have indicated that mice demonstrate humoral immunity and cellular immunity after *L. casei* supplementation (Song et al., 2018). We have previously reported that *L. casei* can regulate the proportion of T lymphocyte subsets and NK cells, thus improving the immune function of rats with alcoholic liver injury or breast cancer (Zhengyan et al., 2017; Yiyun et al., 2019). The findings of our present study indicate that *L. casei* supplementation significantly decreased the percentage of CD8^+^ lymphocytes and NK cells, while the CD4^+^/CD8^+^ ratio was significantly increased. This suggests that the protective effect of *L. casei* on alcohol plus iron‐induced liver injury may be related to the proportion of CD4^+^ and CD8^+^ T lymphocyte subsets and NK cells regulated by *L. casei*.

Previous studies have revealed that enterogenic endotoxin‐mediated inflammation is involved in the progression of alcoholic liver injury. Xiao et al. have demonstrated that treatment with rice bran phenolic extract represses the alcohol‐induced trigger of the hepatic endotoxin‐TLR4‐NF‐κB pathway, followed by mitigated liver inflammation (Xiao & Zhang, 2020). Perea et al. have suggested that treatment with pentraxin‐3 attenuates lipopolysaccharide (LPS)‐induced liver injury and inflammation (Perea et al., 2017). Moreover, iron overload‐induced inflammation causes further damage to the liver tissue (Preziosi et al., 2017). Our previous studies have revealed that *L. casei* improves intestinal injury induced by acrylamide in rats and D‐galactose in aging mice (JiaH Fang et al., 2016; Tianjiao et al., 2018). In the present study, TEM and tracer experiments showed that *L. casei* supplementation could effectively repair the intestinal mucosal barrier damage induced by co‐exposure to alcohol and iron in rats. We observed that the intestinal leakage was significantly alleviated, further confirmed by the serum endotoxin test. Xiao et al. have reported that the phenolic extract of lychee pulp phenolic improves the intestinal barrier function and decreases serum endotoxin levels (Xiao et al., 2017). Subsequently, Western blotting analysis of TLR4, MyD88, NF‐κB p65, and TNF‐α in liver tissue revealed that *L. casei* significantly downregulated the expression of these proteins. Serological tests further confirmed that *L. casei* supplementation inhibited the release of TNF‐α and IL‐1β. Li et al. have reported that ethanol exposure could induce microglia‐mediated neuroinflammation through TLR4 activation and elevated TNF‐α and IL‐1β in rats (Li et al., 2019). The TLR4 MyD88 signaling pathway plays a vital role in the inflammatory response (Ikebe et al., 2009; Takeda et al., 2003). Based on the above evidence, we speculate that the inhibition of inflammation by suppressing the enterogenic endotoxin‐mediated TLR4 signaling pathway could be one of the possible protective mechanisms of *L. casei* on liver injury in rats induced by the synergistic interaction between alcohol and iron.

## 5 CONCLUSIONS

In summary, our findings demonstrate that *L. casei* supplementation could effectively improve liver injury induced by the synergistic interaction between alcohol and iron. The underlying mechanism may involve improved immunity and inhibition of enterogenic endotoxin‐mediated inflammation. These findings provide a scientific basis for developing novel treatment strategies for alcoholic liver disease with coexisting iron overload.

## CONFLICTS OF INTEREST

The authors declare no conflicts of interest.
